# Evaluation of Microfilaremic Individuals after Mass Drug Treatment with Ivermectin, Diethylcarbamazine, and Albendazole for Lymphatic Filariasis in Papua New Guinea

**DOI:** 10.4269/ajtmh.24-0382

**Published:** 2025-03-25

**Authors:** Joycelyn Salo, Simon Westby, Ronnie Wakol, Nelly Sanuku, Krufinta Bun, Moses Laman, Christopher L. King

**Affiliations:** ^1^ Papua New Guinea Institute of Medical Research, Goroka, Papua New Guinea;; ^2^ Center for Global Health and Diseases, Case Western Reserve University School of Medicine, Cleveland, Ohio;; ^3^ Veterans Affairs Medical Center, Cleveland, Ohio

## Abstract

After mass drug administration (MDA) for lymphatic filariasis, which involved a single coadministered dose of ivermectin plus diethylcarbamazine and albendazole (IDA), concerns arose regarding individuals who remained microfilaremic. This situation raised questions about the efficacy of the drugs and whether some individuals had not ingested them. In East New Britain Province, Papua New Guinea (PNG), where 81.7% of the population received IDA, 10 individuals were found to have microfilaremia 12 months after the first round of MDA in an area that had a high baseline of microfilaremia (*n* = 29 microfilariae [Mf] positive pre-MDA). Of these 10 individuals, 7 reported having taken the IDA medication. When Mf detection was repeated 18 months later, all 10 individuals remained Mf positive. Additionally, three more Mf-positive household members were identified, and they also reported taking the IDA. These Mf-positive individuals were then retreated with IDA under direct observation. At 7 and/or 14 months after retreatment, all initially Mf-positive individuals, except for one, were found to be Mf free. Upon further questioning, it was revealed that all but one individual admitted to not taking the initial MDA. Thus, IDA effectively clears Mf in this region of PNG, and the persistent microfilaremia after MDA is primarily because of individuals failing to take the medications as prescribed.

## INTRODUCTION

Lymphatic filariasis (LF) is a parasitic infection caused by the nematodes *Wuchereria bancrofti*, *Brugia malayi*, and *Brugia timori*. The immature worms, known as microfilariae (Mf), are transmitted between humans by mosquitoes. The parasite matures in the lymphatic system, resulting in lymphatic dysfunction, acute lymphangitis, and lymphadenitis, which can progress to disabling lymphedema and hydroceles. Lymphatic filariasis infects approximately 51.4 million people worldwide; an estimated 657 million people in 39 countries remain at risk.^1^

Using evidence that a triple-drug regimen (ivermectin plus diethylcarbamazine and albendazole [IDA]) showed greater efficacy than the standard two-drug regimens,^2^^–^^4^ WHO recommended IDA to accelerate the global elimination of LF.^5^ Two rounds of IDA instead of five rounds are recommended with high coverage (>65%). Ivermectin plus diethylcarbamazine and albendazole is recommended for settings where onchocerciasis is not endemic, areas still needing to start mass drug administration (MDA) as part of the Global Program to Eliminate Lymphatic Filariasis, and areas that have provided fewer than four effective rounds of MDA or MDA with suboptimal (≤65%) coverage. Since its introduction, 20 countries have adopted IDA in at least one LF-endemic district.^1^

Ivermectin plus diethylcarbamazine and albendazole shows >92% complete and sustained Mf clearance in most settings after less than or equal to two rounds.^3^^,^^6^^–^^9^ However, reports of reduced IDA efficacy in Fiji, Samoa, and Cote d’Ivoire have raised concerns.^2^^,^^10^ There are several possible reasons for the reduction in IDA efficacy: 1) failure to take or swallow the medications, 2) differences in drug metabolism, 3) reinfection, or 4) variation in parasite susceptibility to the medication.

Papua New Guinea (PNG) is a western Pacific nation with an estimated population of 11.8 million split across 22 provinces, with 14 provinces considered endemic for LF. In PNG, *W. bancrofti* is the causative nematode of LF, with *Anopheles* mosquitos as the primary vector.^11^ In 2021, New Ireland became the first PNG province to enter postsurveillance after four rounds of MDA: three with diethylcarbamazine plus albendazole and the last with IDA. East New Britain Province (ENBP) became the second province targeted for LF elimination, conducting IDA MDAs in 2019 and 2022. Here, we examined whether microfilaremia in identified individuals after one round of IDA MDA with high epidemiological coverage in ENBP, PNG resulted from drug failure or participants not ingesting the medications.

## MATERIALS AND METHODS

### Study site and population.

**Figure 1.**
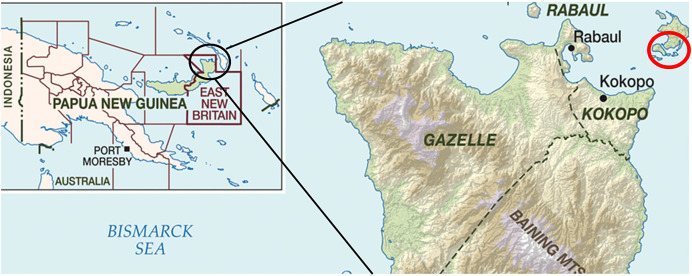
East New Britain Province. Study villages in the Duke of York (DoY) Islands, Kokopo District, are in the red circle in upper right corner.

The study occurred in ENBP, an island province of PNG with an estimated population of 400,000. ENBP has four districts—Kokopo, Rabaul, Gazelle, and Pomio (Figure 1). Kokopo and Rabaul districts are more developed, with access to electricity, water, and paved roads. A baseline LF prevalence survey was conducted September and October 2019 using a mixture of population proportionate sampling and purposeful sampling to select 49 villages across ENBP, sampling roughly equal numbers of children 6–9 years of age and individuals older than 10 years of age.^12^ The mean overall circulationg filarial antigne (CFA) positivity was 5.0% (95% CI: 4.4–5.7, *n* = 213/4,252), and Mf positivity was 1.2% (95% CI: 0.9–1.6, *n* = 52/4,252). Infection levels were highly heterogeneous. Villages reached 37% CFA prevalence and 22% Mf positivity.^12^ Pre-MDA, 29 of the Mf-positive individuals resided in villages surveyed in Duke of York (DoY) Islands, with one village, Utuan, having 22 Mf-positive participants.

The specific sites for this study were three villages identified as having Mf prevalence >1% during the monitoring and evaluation (M&E) conducted after the first round of ivermectin plus diethylcarbamazine and albendazole mass drug administration (MDA1). For this M&E, model-based geostatistics were used to select 24 ENBP villages with a high probability of CFA prevalence >2%.^12^ An additional 23 villages were resampled based on >2% CFA, totaling 47 villages as previously described.^12^ Household members were sampled from a random sample of households until 100 participants were recorded per village.

In total, *n* = 4,611 were sampled, with an overall CFA prevalence of 8.1% (95% CI: 7.3–8.9, *n* = 372/4,611) and an Mf prevalence of 0.33% (95% CI: 0.21–0.42, *n* = 15/4,611).^12^ Six villages with Mf prevalence >1% were identified: three in the Wide Bay area of Pomio district and three in the DoY islands, Kokopo (Figure 1). For accessibility and convenience, the three DoY island sites—Utuan, Karawara, and Kabatirai—were selected for this follow-up study.

The DoY islands are a collection of 13 islands located 25 kilometers northeast of Kokopo town. They are low lying, flat, and covered in tropical forests, with an estimated population of 14,750. Most residents are subsistence farmers and/or fishermen. The baseline Mf prevalences in Utuan, Karawara, and Kabatirai were 21.8%, 7.3%, and 2.0%, respectively, falling to 7.0%, 2.0%, and 1.0%, respectively, after MDA1.^12^

The study population was individuals from these three DoY villages identified as microfilaremic during the post-MDA1 M&E.

### Timeline of treatment, retreatment, and follow-up.

**Figure 2.**
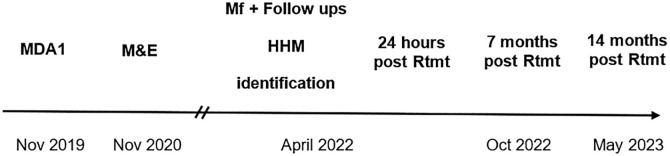
Timeline of treatment in East New Britain Province. HHM = household member; MDA1 = first round of ivermectin plus diethylcarbamazine and albendazole mass drug administration; M&E = monitoring and evaluation; Mf = microfilariae; Nov = November; Oct = October; Rtmt = retreatment.

First round of ivermectin plus diethylcarbamazine and albendazole mass drug administration (“treatment”) occurred in ENBP in November 2019 (Figure 2). It consisted of IDA distributed by health center workers and temporary staff to villages within each catchment area. Dosing determinations were made using a standard dosing poll to approximately ivermectin 200 µg/kg and diethylcarbamazine 6 mg/kg.^13^ Albendazole was given as one 400-mg tablet. The PNG National Department of Health provided the drugs as part of the drug donation program through the Global Program to Eliminate Lymphatic Filariasis. Coverage reported by the ENBP health authority and the PNG National Department of Health was 81.7%, with 93.3% reported coverage in Kokopo district.^12^ An independent coverage survey conducted in December 2019, where population proportionate sampling was used to select 10 local-level government units (clusters) and used again within each cluster to select villages, surveyed 45 villages and yielded similarly high coverage rates of 88.3% (95% CI: 87.1–89.67, *n* = 2,295/2,598) overall reported taking MDA1.^12^

The post-MDA1 M&E survey occurred from November 2020 to January 2021 (Figure 2). As part of the initial survey, all individuals were questioned as to whether they had taken the drugs. Because of the coronavirus disease 2019 (COVID-19) pandemic, the second round of MDA was delayed. In April 2022, those individuals residing in Utuan, Karawara, and Kabatirai identified as Mf positive in November 2020 were revisited to see if they remained Mf positive. At that time, we also examined whether house members of each Mf-positive index case were infected with LF. Questionnaires were conducted face to face and one on one by native Tok Pisin speakers at this repeat follow-up, and individuals were asked about whether they had taken the drugs previously. At the time of questioning, we had the repeat Mf results and provided these results to them, including Mf-positive household members. All individuals with persistent microfilaremia, including positive household contacts, underwent directly observed treatment with IDA (“retreatment”), with dosing as per MDA1. Participants were followed up at 24 hours, 7 months, and 14 months after retreatment to determine whether they cleared microfilaremia.

### LF infection parameters.

Finger-prick blood was collected during the day (between 0600 and 2130) and tested for LF infection using the Alere filariasis test strip (FTS), a rapid test for circulating filarial antigenemia (Alere, Scarborough, ME). Those with a positive FTS result had venous blood collected between 2130 and 2330 into an ethylenediaminetetraacetic acid vacutainer. These samples were transferred in a cool box with ice packs and stored overnight at 4°C before Mf testing using membrane filtration (5 µM; Nuclepore Corporation, Cambridge, MA) of 1 mL blood.^3^ Two microscopists independently read each Giemsa-stained filter to assess Mf counts. FTS and Mf filtration were repeated 24 hours, 7 months, and 14 months after retreatment. If there was discordance between the two readings, a third reader read the slide.

## STATISTICAL ANALYSES

Means, SDs, and statistical tests comparing pre- and posttreatment Mf counts used Microsoft Excel (Microsoft Corp, Redmond, WA).

## RESULTS

### Post-MDA1 M&E and initial follow-up.

During the post-MDA1 M&E, we identified 15 Mf-positive subjects, 10 of whom resided in the selected DoY island villages—7 on Utuan, 2 on Karawara, and 1 on Kabatirai (Table 1). The remaining five, not further discussed in this paper, were in Pomio district.^12^ Of the 10 individuals in the DoY islands, 50% were female, and the average age was 42.4 years old. The Mf concentrations ranged from 33 to 5,785 Mf/mL, with a mean of 882 (±1,747) Mf/mL. Twenty-eight months after MDA1, all 10 individuals remained microfilaremic, with a mean of 469 (±692) Mf/mL and a range of 1–2,062 Mf/mL. Seven of the 10 showed a reduction in Mf concentration at 28 months compared with the 12-month survey, with 6 of those 7 showing a decrease of greater than 60%.

**Table 1:** Impact of retreatment on the Mf-positive individuals identified after the MDA1

Demographics				Reported Swallowing MDA1		Mf Concentration (Mf/mL)				
Index	Village	Age (years)	Sex	Nov 2020	Apr 2022	12 Months Post-MDA1	28 Months Post-MDA1	24 Hours Post-Rtmt	7 Months Post-Rtmt	14 Months Post-Rtmt
1	Karawara	48	M	Yes	NA	166	10	0	NA	0
2	Karawara	53	M	Yes	No	307	9	0	0	0
3	Utuan	30	F	No*	No	374	255	1	0	0
4	Utuan	45	F	Yes	No	42	1	0	NA	4
5	Utuan	25	M	Yes	No	590	932	4	0	0
6	Utuan	50	M	Yes	No	33	48	0	0	0
7	Utuan	56	F	Yes	Yes	888	1,126	2	0	NA
8	Utuan	33	F	No*	No	33	10	0	0	NA
9	Utuan	36	F	Yes	No	5,785	2,062	4	0	0
10	Kabatirai	48	M	No	No	598	239	1	0	0

Apr = April; F = female; M = male; MDA1 = first round of ivermectin plus diethylcarbamazine and albendazole mass drug administration; Mf = microfilariae; NA = not applicable; Nov = November; Rtmt = retreatment.

* This individual did not take MDA1 because she was pregnant.

A year after MDA1, 7 of the 10 microfilaremic follow-up individuals reported receiving and swallowing all of the drugs (Table 1). None reported swallowing only some of the drugs. Two women did not take the drugs because they were pregnant (an exclusion criterion for receiving IDA MDA). When these questions were reasked 28 months after MDA1, only 1 of the 10 insisted that they had taken all of the MDA1 medications. Eight reported not taking any of MDA1, and one was unavailable. These questions were asked after individuals were retested for the presence of Mf, and the results were provided to them.

### Retreatment and follow-up.

Table 1 displays the Mf counts after observing the retreatment of 10 individuals with IDA. Twenty-four hours after retreatment, five participants were amicrofilaremic, and the remaining five had very low Mf concentrations, with a mean of 2.4 (±1.6) Mf/mL. At 7 months after retreatment, eight participants were amicrofilaremic, with data unavailable for the remaining two, both of whom had been amicrofilaremic at 24 hours. At 14 months, eight were amicrofilaremic, with one participant amicrofilaremic at 24 hours and unavailable at 7 months but Mf positive at 14 months (4 Mf/mL).

### Household follow-up.

Twenty-eight household members of 9 of the 10 Mf-positive individuals described in Table 1 were surveyed (Table 2). Of these, seven were found to be FTS positive, and three were Mf positive. At 24 hours after treatment, two of the three Mf-positive individuals had become amicrofilaremic, and one of the three Mf-positive individuals was microfilaremic with 4 Mf/mL. At 7 months, two participants, including the participant who was microfilaremic at 24 hours, had become amicrofilaremic. These two remained amicrofilaremic at 14 months. The remaining participant was unavailable for testing at 7 and 14 months.

**Table 2:** Lymphatic filariasis infections in HH members of Mf-positive individuals after the first round of ivermectin plus diethylcarbamazine and albendazole mass drug administration

Demographics			Infection Status of HH Members		Mf Concentrations (Mf/mL)			
Index Participant	No. of HH Members Surveyed	Age of HH Members (years), Median	No. FTS Positive	No. Mf Positive	Pre-Rtmt	24 Hours Post-Rtmt	7 Months Post-Rtmt	14 Months Post-Rtmt
1	5	11	0	0	–	–	–	–
2	3	43	1	1	2,027	4	0	0
3	6	25	1	0	–	–	–	–
4	1	33	1	1	2,805	0	0	0
5	3	56	1	0	–	–	–	–
6	4	21	3	1	69	0	NA	NA
7	1	39	0	–	–	–	–	–
8	3	10	0	–	–	–	–	–
9	0	–	–	–	–	–	–	–
10	2	34	0	–	–	–	–	–

FTS = filariasis test strip; HH = household; Mf = microfilariae; NA = not applicable; Rtmt = retreatment.

## DISCUSSION

Traditionally, transmission interruption is assessed using the transmission assessment survey after five or more rounds of LF MDA. However, after the introduction of IDA, which can more efficiently reduce Mf in a population, WHO introduced interim guidance in 2019 suggesting that transmission interruption could be assessed using thresholds based on the percentage of Mf-positive adults after just two effective (≥65% coverage) rounds of IDA MDA.^14^

Since the introduction of IDA in 2018, impact surveys conducted in several countries, including Tome and Principe, Timor Leste, Laos, Kenya, and others, have indicated the success of IDA, with MDA no longer needed.^1^ However, some countries, such as American Samoa, Samoa, Fiji, and parts of India, have not yet achieved the recommended end point of interrupting transmission after two rounds of IDA. Possible reasons for these failures include higher pretreatment LF endemicity, more efficient mosquito vectors, overreporting of drug coverage, poor use of bed nets, underdosing, and variations in parasite susceptibility to IDA.^15^^–^^17^

It has been suggested that IDA may have a reduced ability to kill or sterilize adult worms in some individuals, leading to persistent cases of Mf positivity. Although small clinical trials in two other areas of PNG have demonstrated over 95% clearance of Mf after a single dose of IDA 1 or more years later, its effectiveness in PNG’s island provinces, within the context of MDA, has not yet been studied.^3^^–^^5^^,^^14^ Our findings indicate that one dose of IDA remains effective for clearing *W. bancrofti* microfilaremia in an island province of PNG. The persistent microfilaremia observed after IDA MDA is attributed to a failure to ingest IDA rather than a failure of the drug itself. One participant who remained Mf positive after retreatment and was initially Mf negative at 24 hours had an Mf concentration of 4 Mf/mL at 14 months (compared with 42 Mf/mL at 1 year after the initial MDA). This persistence may have been because of reinfection as this participant was from Utuan, the village with the highest Mf prevalence before the first MDA where Mf prevalence was 7% after the first round of MDA.

This study also demonstrates the utility of repeated compliance questionnaires, highlighting that questionnaires undertaken soon after an IDA MDA or as part of an official M&E may yield different results than answers a year or two later, especially when presented with their Mf results. One explanation for this finding could be poor recollection. However, these results could also indicate that there may be social pressure to report compliance with a treatment meant to benefit the community, such as an MDA, when that individual may not have wished to take the medication.^15^ Some individuals may have failed to ingest all of the pills, accounting for a partial reduction in Mf levels in some individuals during MDA. However, all of the participants in this study reported ingesting all of the pills.

Household surveys conducted during this study identified several previously unrecorded and untreated microfilaremic individuals in the households of follow-ups. This highlights the importance of household testing in active case finding after an IDA MDA and the concept of household clustering in LF epidemiology.^15^^,^^16^

There were several limitations to this study. Some participants were unavailable for follow-up at different times. This was somewhat expected as many of the inhabitants of the DoY islands are involved in subsistence farming on islands other than their residences, which may require them to leave early in the morning and return only in the evening. Additionally, those who work in urban areas on the main island may also be unavailable for treatment during MDA. Furthermore, because of the global COVID-19 pandemic, there was an unforeseen delay between the first and second rounds of MDA in ENBP, with the second round taking place in April of 2022.

## CONCLUSION

In conclusion, this study discovered that a single dose of IDA effectively cleared *W. bancrofti* microfilaria in an island province in PNG. It also found that persistent microfilaremia after IDA MDA is because of a failure to ingest IDA rather than drug failure. It emphasizes the importance of high epidemiological coverage and ensuring drug ingestion during LF MDAs to reduce the risk of persistent microfilaremia cases.

## Supplemental Materials

10.4269/ajtmh.24-0382 Supplemental Materials
