# Supplementary material for: Evaluation of Microfilaremic Individuals after Mass Drug Treatment with Ivermectin, Diethylcarbamazine, and Albendazole for Lymphatic Filariasis in Papua New Guinea
Source: Am J Trop Med Hyg. 2025 Mar 25;112(6):1235–9. doi: 10.4269/ajtmh.24-0382 (PMC12139549; doi:10.4269/ajtmh.24-0382)
Supplement: Supplemental Materials [file tpmd240382.SD1.pdf]

Supplementary Tables 1. LF infection parameters pre-MDA

1A. Total CFA and MF prevalence across 49 villages pre-MDA

District	Village	N	Female (N)	Female %	CFA (N)	CFA % (95% CI)	MF (N)	MF % (95% CI)
Kokopo	***Balanataman***	***74***	***41***	***55.41***	***0***	***-***	***0***	
	Ganai	83	50	60.24	4	4.82 (1.3-11.9)	0	
	Kababiai (Doy)	***97***	***51***	***52.58***	***2***	***2.06 (0.3-7.3)***	***1***	***1.03 (0.03-5.6)***
	Kabilomo (Doy)	***83***	***51***	***61.45***	***1***	***1.2 (0.03-6.5)***	***0***	
	Karawara (Doy)	*82*	*49*	*59.76*	*22*	*26.83 (17.6-37.8)*	*6*	*7.32 (2.7-15.3)*
	Malakuna	***100***	***49***	***49***	***0***	***-***	***0***	
	Palavirua	***79***	***42***	***53.16***	***1***	***1.27 (0.03-6.9)***	***0***	
	Ralubang	***79***	***46***	***58.97***	***0***	***-***	***0***	
	Utuwan (Doy)	*101*	*66*	*65.35*	*37*	*36.63 (27.3-46.8)*	*22*	*21.78 (14.2-31.1)*
	Vunamami2	***89***	***55***	***61.8***	***0***	***-***	***0***	
	Vunatagia	***90***	***43***	***47.78***	***2***	***2.22 (0.3-7.8)***	***0***	
Gazelle	***Bitakapuk3***	***74***	***46***	***62.16***	***0***	***-***	***0***	
	Kadaulung	***96***	***47***	***48.96***	***0***	***-***	***0***	
	Karo	99	61	61.62	10	10.1 (4.9-17.8)	0	
	Kikitabu	***86***	***43***	***50***	***0***	***-***	***0***	
	Lan	*101*	*63*	*62.38*	*3*	*2.97 (0.6-8.4)*	*0*	
	Matanakunai	86	49	56.98	3	3.49 (0.7-9.9)	0	
	Mobilim	82	41	50	3	3.66 (0.8-10.3)	0	
	Napapar1	***91***	***50***	***54.95***	***5***	***5.49 (1.8-12.4)***	***3***	***3.3 (0.7-9.3)***
	Navui	32	13	40.63	0	-	0	
	Ratavul	***97***	***48***	***49.48***	***0***	***-***	***0***	
	Takekel	***102***	***55***	***53.92***	***1***	***0.98 (0.02-5.3)***	***0***	
	Tavilo	***85***	***43***	***50.59***	***0***	***-***	***0***	
	Vunairoto	***72***	***41***	***56.94***	***0***	***-***	***0***	
	Vunapalinding1	***83***	***49***	***59.04***	***5***	***6.02 (1.9-13.5)***	***2***	***2.41 (0.3-8.4)***
	Warakindam	101	60	59.41	8	7.92 (3.5-15.0)	1	0.99 (0.03-5.4)
	Watwat	***105***	***56***	***53.33***	***2***	***1.9 (0.2-6.7)***	***0***	

	Yayem	***95***	***47***	***49.47***	***0***	***-***	***0***	
Pomio	Dadul	89	50	56.18	0	-	0	
	Gar	94	52	55.32	2	2.13 (0.3-7.5)	0	
	Hoiya	***85***	***46***	***54.12***	***23***	***27.06 (17.9-37.8)***	***3***	***3.53 (0.7-9.9)***
	Illi	59	30	50.85	3	5.08 (1.1-14.2)	0	
	Katap	***64***	***33***	***52.38***	***0***	***-***	***0***	
	Kaukum	88	48	54.55	16	18.18 (10.8-27.8)	2	2.27 (0.3-7.9)
	Kavudemki	60	32	53.33	10	16.67 (8.3-28.5)	1	1.67 (0.04-8.9)
	Lamarian	*94*	*51*	*54.26*	*5*	*5.32 (1.8-11.9)*	*2*	*2.13 (0.3-7.5)*
	Lat	*114*	*65*	*57.02*	*16*	*14.04 (8.2-21.8)*	*1*	*0.88 (0.02-4.8)*
	Marunga	***94***	***51***	***54.26***	***1***	***1.06 (0.03-5.8)***	***0***	
	Masarau	***89***	***41***	***46.07***	***7***	***7.87 (3.2-15.5)***	***3***	***3.37 (0.7-9.5)***
	Mazo	119	70	58.82	2	1.68 (0.2-5.9)	0	
	Pulpul	101	55	54.46	3	2.97 (0.62-8.4)	0	
	Riete	103	54	52.43	2	1.94 (0.24-6.8)	2	1.94 (0.2-6.8)
	Sivauna	***66***	***33***	***50***	***2***	***3.03 (0.4-10.5)***	***1***	***1.52 (0.04-8.2)***
	Tokai	89	47	52.81	8	8.99 (3.9-16.9)	1	1.12 (0.03-6.1)
Rabaul	***Livuan***	***102***	***55***	***54.46***	***1***	***0.98 (0.02-5.3)***	***0***	
	Malaguna3	***94***	***61***	***64.89***	***1***	***1.06 (0.03-5.8)***	***0***	
	Matupit	68	44	64.71	0	-	0	
	Tavui1	64	34	53.13	0	-	0	
	Volavolo	***72***	***39***	***54.17***	***2***	***2.78 (0.34-9.7)***	***1***	***1.39 (0.04-7.5)***

Bold lettered villages are randomly selected by population proportionate sampling (PPS)

Italicized villages are 5 purposively selected by the East New Britain LF task.

Table 2A. Summary of LF infection parameters in children and adults pre-MDA

		6-9 years					≥10 years			
District	Population	CFA (N)	CFA % (95% CI)	MF (N)	MF % (95% CI)	N	CFA (N)	CFA % (95% CI)	MF (N)	MF % (95% CI)
Kokopo	480	23	4.79 (3.1- 7.1)	5	1.04 (0.3-2.4)	827	30	3.6 (2.5-5.1)	6	0.7 (0.3-1.6)
Gazelle	660	10	1.52 (0.7-2.8)	0	-	477	46	9.6 (7.2-12.7)	24	5.0 (3.3-7.4)
Pomio	581	4	0.69 (0.2-1.8)	0	-	827	96	11.6 (9.5-13.9)	16	1.9 (1.1-3.1)
Rabaul	185	0	-	0	-	215	4	1.9 (0.5-4.7)	1	0.5 (0.0-2.6)
**Total**	**1906**	**37**	**1.9 (1.4-2.7)**	**5**	**0.3 (0.1-0.6)**	**2346**	**176**	**7.5 (6.5-8.6)**	**47**	**2.0 (1.5-2.7)**

2B. Total CFA and MF prevalence in 6- 9 year age-group pre-MDA

District	Villages	N	F	%F	CFA (N)	CFA % 95% CI	MF (N)	MF %
Kokopo	Balanataman	48	23	47.92	0			
	Ganai	38	15	39.47	0			
	Kababiai	46	22	47.83	0			
	Kabilomo	35	19	54.29	0			
	Karawara	51	24	47.06	11	21.57 (11.3-35.3)	1	1.96 (0.1-10.5)
	Malakuna	48	18	37.50	0			
	Palavirua	36	14	38.89	1	2.78 (0.1-14.5)		
	Ralubang	35	17	48.57	0			
	Utuwan	51	29	56.86	11	21.57 (11.3-35.3)	4	7.84 (2.2-18.9)
	Vunamami2	61	36	59.02	0			
	Vunatagia	31	10	32.26	0			
Gazelle	Bitakapuk3	29	19	65.52	0			
	Kadaulung	16	8	50.00	0			
	Karo	48	27	56.25	2	4.17 (0.5-14.3)		
	Kikitabu	36	12	33.33	0			

	Lan	50	28	56.00	1	2 (0.1-10.7)		
	Matanakunai	31	15	48.39	0			
	Mobilim	26	15	57.69	0			
	Napapar1	41	21	51.22	1	2.44 (0.1-12.9)		
	Navui	32	13	40.63	0			
	Ratavul	49	22	44.90	0			
	Takekel	52	29	55.77	1	1.92 (0.1-10.3)		
	Tavilo	27	13	48.15	0			
	Vunairoto	34	17	50.00	0			
	Vunapalinding1	50	26	52.00	3	6 (1.3-16.6)		
	Warakindam	53	28	52.83	2	3.77 (0.5-12.9)		
	Watwat	51	23	45.10	0			
	Yayem	35	19	54.29	0			
Pomio	Dadul	23	7	30.43	0			
	Gar	43	20	46.51	1	2.33 (0.1-12.3)		
	Hoiya	28	14	50.00	2	7.14 (0.88-23.5)		
	Illi	19	8	42.11	0			
	Katap	17	9	52.94	0			
	Kaukum	39	18	46.15	1	2.56 (0.1-13.5)		
	Kavudemki	26	9	34.62	0			
	Lamarian	42	17	40.48	0			
	Lat	34	17	50.00	0			
	Marunga	34	16	47.06	0			
	Masarau	37	16	43.24	0			
	Mazo	66	30	45.45	0			
	Pulpul	50	21	42.00	0			
	Riete	43	16	37.21	0			
	Sivauna	29	15	51.72	0			
	Tokai	51	27	52.94	0			
Rabaul	Livuan	50	25	50.00	0			

	Malaguna3	46	26	56.52	0			
	Matupit	30	19	63.33	0			
	Tavui1	28	13	46.43	0			
	Volavolo	31	13	41.94	0			

2C. Total CFA and MF prevalence in =>10 year age-group at pre-MDA

District	Village	N	F	%F	CFA (N)	CFA % (95% CI)	MF (N)	MF % (95% CI)
Kokopo	Balanataman	26	18	69.23	0			
	Ganai	45	35	77.78	4	8.89 (2.5-21.2)	0	
	Kababiai	51	29	56.86	2	3.92 (0.5-13.5)	1	1.96 (0.1-10.5)
	Kabilomo	48	32	66.67	1	2.08 (0.1-11.1)		
	Karawara	31	25	80.65	11	35.48 (19.2-54.6)	5	16.13 (5.5-33.7)
	Malakuna	52	31	59.62	0			
	Palavirua	43	28	65.12	0			
	Ralubang	44	29	65.91	0			
	Utuan	50	37	74.00	26	52 (37.4-66.3)	18	36 (22.9-50.8)
	Vunamami2	28	19	67.86				
	Vunatagia	59	33	55.93	2	3.39 (2-3.4)	0	
Gazelle ‘	Bitakapuk3	45	27	60.00				
	Kadaulung	80	39	48.75				
	Karo	51	34	66.67	8	15.69 (7.0-28.6)		
	Kikitabu	50	31	62.00				
	Lan	51	35	68.63	2	3.92 (0.5- 13.5)	0	
	Matanakunai	55	34	61.82	3	5.45 (1.1-15.1)	0	
	Mobilim	56	26	46.43	3	5.36 (1.1-14.9)	0	
	Napapar1	50	29	58.00	4	8 (2.2-19.2)	3	6 (1.3-16.6)
	Navui	0	0	-				
	Ratavul	48	26	54.17				
	Takekel	50	26	52.00				
	Tavilo	58	30	51.72				

	Vunairoto	38	24	63.16				
	Vunapalinding1	33	23	69.70	2	6.06 (2-6.1)	2	6.06 (0.7-20.2)
	Warakindam	48	32	66.67	6	12.5 (4.7-25.3)	1	2.08 (0.1-11.1)
	Watwat	54	33	61.11	2	3.7 (0.5-12.8)	0	
	Yayem	60	28	46.67				
Pomio	Dadul	66	43	65.15				
	Gar	51	43	84.31	1	1.96 (0.1-10.5)	0	
	Hoiya	57	32	56.14	21	36.84 (24.5-50.7)	3	5.26 (1.1-14.6)
	Illi	40	22	55.00	3	7.5 (1.6-20.4)	0	
	Katap	47	24	51.06				
	Kaukum	49	30	61.22	15	30.61 (18.3-45.4)	2	4.08 (0.5-13.9)
	Kavudemki	34	23	67.65	10	29.41 (15.1-47.5)	1	2.94 (0.1-15.3)
	Lamarian	52	34	65.38	5	9.62 (3.2-21.0)	2	3.85 (0.5-13.2)
	Lat	80	48	60.00	16	20 (11.9-30.4)	1	1.25 (0.0-6.8)
	Marunga	60	35	58.33	1	1.67 (0.0-8.9)	0	
	Masarau	52	25	48.08	7	13.46 (5.6-25.8)	3	5.77 (1.2-15.9)
	Mazo	53	40	75.47	2	3.77 (0.5-12.9)	0	
	Pulpul	51	34	66.67	3	5.88 (1.2-16.2)	0	
	Riete	60	38	63.33	2	3.33 (0.4-11.5)	2	3.33 (0.4-11.5)
	Sivauna	37	18	48.65	2	5.41 (0.7-18.2)	1	2.7 (0.1-14.2)
	Tokai	38	20	52.63	8	21.05 (9.6-37.3)	1	2.63 (0.1-13.8)
Rabaul	Livuan	52	30	57.69	1	1.92 (0.1-10.3)	0	
	Malaguna3	48	35	72.92	1	2.08 (0.1-11.1)	0	
	Matupit	38	25	65.79				
	Tavui1	36	21	58.33				
	Volavolo	41	26	63.41	2	4.88 (0.6-16.5)	1	2.44 (0.1-12.9)

2D. Total CFA and MF prevalence in =>10-17 year age-group at pre-MDA

District	Village	N	F	%F	CFA (N)	CFA % (95% CI)	MF (N)	MF % (95% CI)
Kokopo	Balanataman	5	1	20.00	0			
	Ganai	14	10	71.43	1	7.14 (0.2-33.9)	0	
	Kababiai	15	6	40.00	0			
	Kabilomo	11	7	63.64	1	9.1 (0.2-41.3)	0	
	Karawara	11	9	81.82	4	36.4 (10.9- 69.2)	3	27.27 (6.0-60.9)
	Malakuna	27	12	44.44	0			
	Palavirua	15	13	86.67	0			
	Ralubang	9	3	33.33	0			
	Utuwan	12	7	58.33	3	25 (5.5-57.2)	2	16.67 (2.1-48.4)
	Vunamami2	4	1	25.00	0			
	Vunatagia	15	10	66.67	0			
Gazelle	Bitakapuk3	13	8	61.54	0			
	Kadaulung	14	8	57.14	0			
	Karo	8	3	37.50	0			
	Kikitabu	13	7	53.85	0			
	Lan	5	3	60.00	0			
	Matanakunai	7	2	28.57	0			
	Mobilim	18	9	50.00	1	5.56 (0.1-27.3)	0	
	Napapar1	15	5	33.33	1	6.67 (0.2-31.9)	0	
	Navui	0	0	-	-			
	Ratavul	11	6	54.55	0			
	Takekel	16	8	50.00	0			
	Tavilo	15	5	33.33	0			
	Vunairoto	11	4	36.36	0			
	Vunapalinding1	5	2	40.00	0			

	Warakindam	15	11	73.33	0			
	Watwat	15	7	46.67	1	6.67 (0.2-31.9)	0	
	Yayem	23	11	47.83	0			
Pomio	Dadul	18	11	61.11	0			
	Gar	16	8	50.00	0			
	Hoiya	19	9	47.37	4	21.05 (6.1- 45.6)	1	5.26 (0.1-26.0)
	Illi	15	9	60.00	1	6.67 (0.2- 31.9)		
	Katap	25	13	52.00	0			
	Kaukum	16	11	68.75	3	18.75 (4.1-45.7)	0	
	Kavudemki	10	4	40.00	2	20 (2.5-55.6)	0	
	Lamarian	11	5	45.45	0			
	Lat	28	17	60.71	0			
	Marunga	16	10	62.50	0			
	Masarau	6	1	16.67	0			
	Mazo	1	1	100.00	0			
	Pulpul	19	12	63.16	0			
	Riete	14	9	64.29	0			
	Sivauna	11	4	36.36	0			
	Tokai	11	5	45.45	0			
Rabaul	Livuan	24	13	54.17	0			
	Malaguna3	5	2	40.00	0			
	Matupit	17	10	58.82	0			
	Tavui1	9	5	55.56	0			
	Volavolo	20	9	45.00	1	5 (0.1- 24.9)	0	

2E. Total CFA and MF prevalence in =>18 years at pre-MDA

District	Village	N	F	%F	CFA (N)	CFA % (95% CI)	MF (N)	MF % (95% CI)
Kokopo	Balanataman	21	17	80.95	0			

	Ganai	31	25	80.65	3	9.68 (2.0-25.8)	0	
	Kababiai	36	23	63.89	2	5.56 (0.7-18.7)	1	2.78 (0.1-14.5)
	Kabilomo	37	25	67.57	0			
	Karawara	20	16	80.00	7	35 (15.4-59.2)	2	10 (1.2-31.7)
	Malakuna	25	19	76.00	0			
	Palavirua	28	15	53.57	0			
	Ralubang	35	26	74.29	0			
	Utuwan	38	30	78.95	23	60.53 (43.4-75.9)	16	42.11 (26.3-59.2)
	Vunamami2	24	18	75.00	0			
	Vunatagia	44	23	52.27	2	4.55 (0.6-15.5)		
Gazelle	Bitakapuk3	32	19	59.38	0			
	Kadaulung	66	31	46.97	0			
	Karo	43	31	72.09	8	18.6 (8.4-33.4)	0	
	Kikitabu	37	24	64.86	0			
	Lan	46	32	69.57	2	4.35 (0.5-14.8)	0	
	Matanakunai	48	32	66.67	3	6.25 (1.3-17.2)		
	Mobilim	38	17	44.74	2	5.26 (0.6-17.8)	0	
	Napapar1	35	24	68.57	3	8.57 (1.8-3.1)	3	8.57 (1.8-23.1)
	Navui	0	0	0.00	0			
	Ratavul	37	20	54.05	0			
	Takekel	34	18	52.94	0			
	Tavilo	43	25	58.14	0			
	Vunairoto	27	20	74.07	0			
	Vunapalinding1	28	21	75.00	2	7.14 (0.9-23.5)	2	7.14 (0.9-23.5)
	Warakindam	33	21	63.64	6	18.18 (6.9-53.5)	1	3.03 (0.1-15.8)
	Watwat	39	26	66.67	1	2.56 (0.1-13.5)		
	Yayem	37	17	45.95	0			
Pomio	Dadul	48	32	66.67	0			
	Gar	35	24	68.57	1	2.86 (0.1-14.9)	0	
	Hoiya	38	23	60.53	17	44.74 (28.6-61.7)	2	5.26 (0.6-17.8)
	Illi	25	13	52.00	2	8 (0.9-26.0)	0	

	Katap	22	11	50.00	0			
	Kaukum	33	19	57.58	12	36.36 (20.4-54.9)	2	6.06 (0.7-20.2)
	Kavudemki	24	19	79.17	8	33.33 (15.6-55.3)	1	4.17 (0.1-21.1)
	Lamarian	41	29	70.73	5	12.2 (4.1-26.2)	2	4.88 (0.6-16.5)
	Lat	52	31	59.62	16	30.77 (18.7-45.1)	1	1.92 (0.1-10.3)
	Marunga	44	25	56.82	1	2.27 (0.1-12.0)	0	
	Masarau	46	24	52.17	7	15.22 (6.3-28.9)	3	6.52 (1.4-17.9)
	Mazo	52	39	75.00	2	3.85 (0.5-13.2)	0	
	Pulpul	32	22	68.75	3	9.38 (1.9-25.0)	0	
	Riete	46	29	63.04	2	4.35 (0.5-14.8)	2	4.35 (0.5-14.8)
	Sivauna	26	14	53.85	2	7.69 (0.9-25.1)	1	3.85 (0.1-19.6)
	Tokai	27	15	55.56	8	29.63 (13.8-50.2)	1	3.7 (0.1-18.9)
Rabaul	Livuan	28	17	60.71	1	3.57 (0.1-18.4)	0	
	Malaguna3	43	33	76.74	1	2.33 (0.1-12.3)	0	
	Matupit	21	15	71.43	0			
	Tavui1	27	16	59.26	0			
	Volavolo	21	17	80.95	1	4.76 (0.12-23.8)	1	4.76 (0.1-23.8)

Table 3.

	Age-range	N	CFA (N)	CFA % (95% CI)	MF (N)	MF PREV
**Female**	6-9	918	21	2.29 (1.42-3.48)	3	0.33 (0.07-0.95)
	10-17	346	15	4.34 (3.43-5.23)	5	1.45 (0.92-1.97)
	>=18	1082	89	8.23 (7.53-8.91)	25	2.31 (1.93-2.68)
**Males**	6-9 years	988	16	1.62 (0.93-2.62)	2	0.20 (0.02-0.73)
	10-17 yrs	307	8	2.61 (1.86-3.35)	1	0.33 (0.05-0.59)
	>=18	611	64	10.47 (9.46-11.49)	16	2.62

(2.09-3.15)

Table 4. Infection parameters in children and adults in PPS selected villages.

	**Total CFA and MF % in randomized selected villages by PPS**					**6-9 years**					**≥10 years**				
**District**	**Population**	**CFA (N)**	**CFA % (95% CI)**	**Mf (n)**	**Mf % (95% CI**	**N**	**CFA (N)**	**CFA % (95% CI)**	**MF (N)**	**Mf % (95% CI)**	**N**	**CFA (N)**	**CFA % (95% CI)**	**MF (N)**	**MF % (95% CI)**
**Kokopo**	691	6	0.87 (0.3-1.9)	1	0.1 (0.0-0.8)	340	1	0.3 (0.0-1.6)	0	-	351	5	1.4 (0.5-3.3)	1	0.3 (0.0-1.6)
**Gazelle**	1072	16	1.5 (0.9-2.4)	5	0.5 (0.2-1.1)	451	5	1.1 (0.4-2.6)	0	-	621	11	1.8 (0.9-3.2)	5	0.8 (0.3-1.9)
**Pomio**	398	33	8.29 (5.8-11.5)	7	1.8 (0.7-3.6)	145	2	1.4 (0.2-4.9)	0	-	253	31	12.3 (8.5-16.9)	7	2.8 (1.1-5.6)
**Rabaul**	400	4	1.0 (0.3-2.5)	1	0.3 (0.0-1.4)	185	0	-	0	-	215	4	1.9 (0.5-4.7)	1	0.5 (0.0-2.6)
**Total**	**2561**	**59**	**2.3 (1.8-2.9)**	**14**	**0.6 (0.3-0.9)**	**1121**	**8**	**0.7 (0.3-1.4)**	**0**	**-**	**1440**	**51**	**3.5 (2.7-4.6)**	**14**	**0.9 (0.5-1.6)**

Table 5. Infection parameters in children and adults in purposively selected villages pre-MDA.

	Total CFA and MF % in purposive selected villages					6-9 years					≥10 years				
**District**	**Popn.**	**CFA + (N)**	**CFA % (95% CI)**	**Mf (N)**	**Mf % (95% CI**	**N**	**CFA (N)**	**CFA % (95% CI)**	**MF (N)**	**MF % (95% CI)**	**N**	**CFA (N)**	**CFA % (95% CI)**	**MF (N)**	**MF % (95% CI)**
Kokopo	266	63	23.7 (18.7-29.3)	28	10.5 (7.1-14.8)	140	22	15.7 (10.1-22.8)	5	3.6 (1.2-8.1)	126	41	32.5 (24.5-41.5)	23	18.3 (11.9-26.1)
Gazelle	415	24	5.8 (3.7-8.5)	1	0.24 (0.0-1.3)	209	5	2.4 (0.8-5.5)	0	-	206	19	9.2 (5.6-14.0)	1	0.5 (0.0-2.7)
Pomio	1010	67	6.6 (5.2-8.4)	9	0.9 (0.4-1.7)	436	2	0.5 (0.1-1.7)	0	-	574	65	11.3 (8.9-14.2)	9	1.6 (0.7-2.9)
Rabaul *	0	0	-	0	-	-	-	-	-		-			-	
**Total**	**1691**	**154**	**9.1 (7.8-10.6)**	**38**	**2.3 (1.6-3.1)**	**785**	**29**	**3.7 (2.5-5.3)**	**5**	**0.6 (0.2-1.5)**	**906**	**125**	**13.8 (11.6-16.2)**	**33**	**3.6 (2.5-5.1)**

*Villages in the Rabaul district were not purposively selected because they were categorized as a low-risk having <2% CFA and mf prevalence in previously surveyed villages.

Table 6. Administrative coverage of IDA in ENBP November 2019

District	Health Facility	Pop 2019	TREATMENT WITH IDA/DA						Total treated	Treatment Coverage /100% pop (%)	Lymphadema present				MDA Coverage %
			Female			Male									
			2-4YRS	5-14YRS	15≦YRS	2-4YRS	5-14YRS	15≦YRS			Han solap	Susu solap	Lek solap	Bol solap	
Kokopo District															
	Bitapaka HC	20535	974	2695	5336	1052	3195	5524	18776	91.4	15	9	29	23	93.9
	Molot HC	8034	293	801	2053	290	857	1865	6159	76.7	0	0	1	1	78.4
	Vatnabara HC	6515	287	761	1814	281	820	1677	5640	86.6	8	5	26	12	90.2
	St.Mary's Hospital	18902	976	2998	6334	1047	3294	7183	21832	115.5	0	0	3	10	117.9
	Butuwin HC	37177	1475	3750	10054	1376	3890	10395	30940	83.2	3	0	8	6	84.3
	Sub-total	91163	4005	11005	25591	4046	12056	26644	83347	91.4	26	14	67	52	93.3
Rabaul District															
	Nonga Hospital	2380	310	1154	2829	346	1288	2619	8546	359.1	0	0	1	0	376.3
	Rabaul UC	16899	324	1312	3045	332	1354	3661	10028	59.3	0	0	7	2	60.7
	Gelegele HC	9178	421	1272	2379	415	1413	2086	7986	87.0	0	0	0	1	90.4
	Sub-total	28457	1055	3738	8253	1093	4055	8366	26560	93.3	0	0	8	3	96.7
Gazelle District															
	Napapar HC	23770	779	2742	5089	917	2992	4998	17517	73.7	2	0	4	2	76.4
	Kerevat RH	36540	1097	3260	6294	1065	3432	7329	22477	61.5	4	4	3	6	63.8
	Raunsepna HC	3481	186	438	668	201	456	700	2649	76.1	0	0	0	0	80.1
	Gaulim HC	10284	657	1507	3296	666	1867	4836	12829	124.7	0	2	1	7	131.4
	Lassul Bay HC	7649	416	1184	2133	500	1425	2685	8343	109.1	4	3	23	22	113.9
	Open Bay HC	6356	353	748	1554	382	877	2378	6292	99.0	5	1	7	8	104.8
	Livuan DC	16107	693	2269	4526	770	2286	4095	14639	90.9	1	2	4	2	91.7
	Vunapaka HC	17653	625	1924	3961	662	2041	4013	13226	74.9	0	0	0	0	75.4
	Tapipipi HC	27384	621	2223	4660	751	2419	4285	14959	54.6	3	0	1	0	56.6
	Paparatava HC	20742	1353	4003	7236	1428	4040	7015	25075	120.9	0	0	0	21	123.9
	Sub-total	169967	6780	20298	39417	7342	21835	42334	138006	81.2	19	12	43	68	83.8
Pomio District															
	Pomio HC	7987	251	773	1398	262	780	1365	4829	60.5	5	3	11	7	64.2
	Matong HC	3103	134	380	651	117	418	697	2397	77.3	0	0	6	3	82.8
	Muela HC	9808	288	702	940	278	730	958	3896	39.7	0	2	1	0	43.6
	Nutuve HC	4419	264	609	936	280	652	907	3648	82.6	0	0	0	0	93.4
	Hoiya HC	2575	129	325	646	151	354	791	2396	93.0	9	1	20	10	97.5
	Guma HC	4612	151	504	828	159	541	782	2965	64.3	0	0	5	1	66.8
	Uvol HC	13492	401	1275	2392	450	1287	2374	8179	60.6	0	0	5	4	65.1
	Warangoi RH	15300	613	1286	2579	581	1588	3134	9781	63.9	1	0	4	6	66.2
	Mungou HC	4823	138	371	906	122	391	1166	3094	64.2	0	0	3	4	67.2
	Marunga HC	5052	191	358	1035	192	423	1402	3601	71.3	1	1	11	0	75.2
	Palmalmal RH	7693	330	897	2528	323	992	3134	8204	106.6	0	1	1	0	112.2
	Viosopuna HC	2754	85	302	543	104	338	514	1886	68.5	0	0	0	0	70.8
	Aona HC	4322	158	372	604	178	395	573	2280	52.8	0	0	1	0	56.8
	Sivaona HC	1041	123	361	716	129	351	819	2499	240.2	0	1	9	0	258.0
	Sub-total	86979	3256	8515	16702	3326	9240	18616	59655	68.6	16	9	77	35	72.8
	Total	376566	15096	43556	89963	15807	47186	95960	307568	81.7	61	35	195	158	84.6

Table 7. Total CFA and MF prevalence across 47 villages at 1 year post-MDA

District	VILLAGE	N	FEMALE (N)	Female %	CFA (N)	CFA % (95% CI)	Mf (N)	Mf %
Kokopo	Ganai	100	69	69	16	16.16 (9.5-24.9)	0	0
	Kababia	100	57	57	6	6 (2.2-12.6)	0	0
	Kabatira	100	60	60	6	6 (2.2-12.6)	1	1 (0.0-5.5)
	Karawara	100	47	47	22	22.28 (14.3-31.4)	2	2 (0.2-7.0)
	Mualim	99	58	59.6	4	4.04 (1.11-10.02)	0	0
	Palpal	101	75	74.26	7	6.93 (2.8-13.8)	0	0
	Ralauna	58	37	63.79	0	0	0	0
	Utuwan	100	57	57	36	36 (26.6-46.2)	7	7 (2.9-13.9)
	Virian	100	63	63	3	3 (0.62-8.5)	0	0
Gazelle	*Kamanaka*	100	57	57	0	0	0	0
	Karo	100	52	52	1	1 (0.03-5.5)	0	0
	Lan	100	54	54	2	2 (0.24-7.04)	0	0
	Matanaku	102	56	54.9	3	2.94 (0.61-8.4)	0	0
	Mobilim	100	35	35	4	4 (1.1-9.9)	0	0
	Napapar1	98	55	56.12	2	2.04 (0.3-7.2)	0	0
	Puktas	100	55	55	4	4 (1.1-9.9)	0	0
	Ragaga	100	61	61	6	6.06 (2.23-12.6)	0	0
	Ulak	99	52	52.53	3	3.03 (0.63-8.6)	0	0
	Vunapala	102	63	61.76	1	0.98 (0.02-5.3)	0	0
	Warakind	100	52	52	7	7 (2.9-13.9)	0	0
	Wuatam	99	25	25.25	2	2.04 (0.3-7.2)	0	0
Pomio	*Awatka*	100	55	55	4	4 (1.1-9.9)	0	0
	Bogotata	103	62	60.19	13	12.75 (6.9-20.9)	0	0
	Buka	98	62	63.27	24	24.49 (16.4-34.2)	0	0
	Bulus	98	55	56.12	19	19.39 (12.1-28.6)	0	0
	Gar	99	62	62.63	0	0	0	0
	Gumgum	100	45	45	6	6 (2.2-12.6)	1	1 (0.03-5.5)
	Hoiya	99	55	55.56	17	17.17 (10.3-26.1)	0	0

	Illi	100	48	48	0	0	0	0
	Ivai	98	74	75.51	6	6.12 (2.3-12.9)	1	1.02 (0.0-5.6)
	Karlai	100	46	46	17	17 (10.2-25.8)	0	0
	Kaukum	100	45	45	16	16 (9.4-24.7)	0	0
	Kavudemk	100	56	56	12	12.12 (6.4-20.0)	0	0
	Kolai	93	51	54.84	4	4.3 (1.2-10.7)	0	0
	Lamarian	99	53	53.54	11	11.11 (5.6-19.01)	0	0
	Lat	160	74	46.25	20	12.5 (7.8-18.7)	1	0.63 (0.0-3.4)
	Long	99	36	36.36	14	14.29 (7.9-22.6)	1	1.01 (0.0-5.5)
	Masarau	77	50	64.94	5	6.49 (2.1-14.5)	0	0
	Milim	99	59	59.6	6	6.06 (2.3-12.7)	0	0
	Muu	99	48	48.48	8	8.08 (3.6-15.3)	0	0
	PomComSc	82	47	57.32	0	0	0	0
	Pulpul	87	55	63.22	6	6.98 (2.6-14.4)	0	0
	Rainut	68	32	47.06	2	2.94 (0.4-10.2)	0	0
	Riete	100	51	51	2	2 (0.2-7.0)	0	0
	Sivauna	96	56	58.33	9	9.47 (4.4-17.1)	0	0
	Tokai	99	58	58.59	16	16.16 (9.5-24.9)	0	0
Rabaul	Tavui	100	58	58	0	0	0	0

Table 7A. Summary of LF Infection parameters of children and adults 1 year post-MDA.

	6-9						≥ 18				
District	N	CFA (N)	CFA % (95% CI)	N	MF (N)	Mf % (95% CI)	N	CFA (N)	CFA % (95% CI)	MF (N)	MF % (95% CI)
Kokopo	231	9	3.9 (1.8-2.3)	231	0	-	627	91	14.5 (11.9-17.6)	10	1.6 (0.8-2.9)
Gazelle	93	1	1.1 (0.0-5.9)	93	0	-	1107	34	3.1 (2.1-4.3)	0	0.00
Pomio	370	10	2.7 (1.3-4.9)	370	0	-	2083	227	10.9 (9.6-12.4)	4	0.2 (0.1-0.5)
Rabaul	1	0	-	1	0	-	99	0	-	0	
**Total**	**695**	**20**	**2.8 (1.8-4.4)**	**695**	**0**	**-**	**3916**	**352**	**9.0 (8.1-9.9)**	**14**	**0.4 (0.2-0.6)**

Table 7B Total CFA and MF prevalence in 6- 9 year age-group post-MDA for each village

District	Village	N	F (N)	F (%)	CFA (N)	CFA % (95% CI)	MF (N)	MF % (95% CI)
Kokopo	Ganai	0						
	Kababia	0						
	Kabatira	47	22	46.81	0	-	0	
	Karawara	47	19	40.43	5	10.64 (3.6-23.1)	0	
	Mualim	48	29	60.42	0			
	Palpal	0						
	Ralauna	0						
	Utuwan	45	26	57.78	4	8.89 (2.5-21.2)	0	
	Virian	44	21	47.73	0		0	
Gazelle	Kamanaka	0						
	Karo	46	21	45.65	0		0	
	Lan	47	28	59.57	1	2.13 (0.1-11.3)	0	
	Matanaku	0						
	Mobilim	0						
	Napapar1	0						

	Puktas	0						
	Ragaga	0						
	Ulak	0						
	Vunapala	0						
	Warakind	0						
	Wuatam	0						
Pomio	Awatka	0						
	Bogotata	21	8	38.10	1	4.76 (0.1-23.8)	0	
	Buka	17	15	88.24	1	5.88 (0.2-28.7)	0	
	Bulus	0						
	Gar	40	22	55.00	0		0	
	Gumgum	0						
	Hoiya	41	19	46.34	2	4.88 (0.6-16.5)	0	
	Illi	0						
	Ivai	0						
	Karlai	0						
	Kaukum	49	20	40.82	3	6.12 (1.3-16.9)	0	
	Kavudemk	0						
	Kolai	43	17	39.53	0	-	0	
	Lamarian	0						
	Lat	53	16	30.19	0	-	0	
	Long	0						
	Masarau	0						
	Milim	11	6	54.55	0	-	0	
	Muu	0						
	PomComSc	0						
	Pulpul	1	1	100.00	0	-	0	
	Rainut	18	8	44.44	0	-	0	
	Riete	0						
	Sivauna	47	28	59.57	3	6.52 (1.4-17.9)	0	
	Tokai	29	13	44.83	0		0	

Rabaul	Tavui	1	1	100.00	0		0	

Table 7C Total CFA and MF prevalence in >18 year age-group post-MDA for each village

District	Village	N	Female (N)	Female (%)	CFA (N)	CFA % (95% CI)	Mf (N)	Mf % (95% CI)
Kokopo	Ganai	100	69	69.00	16	16.16 (9.5-24.9)		
	Kababia	100	57	57.00	6	6 (2.2-12.6)		
	Kabatira	53	38	71.70	6	11.32 (4.3-23.0)	1	1.89 (0.1-10.1)
	Karawara	53	28	52.83	17	32.69 (20.3-47.1)	2	3.77 (0.5-12.9)
	Mualim	51	30	58.82	4	7.84 (2.2-18.9)		
	Palpal	101	75	74.26	7	6.93 (2.8-13.8)		
	Ralauna	58	37	63.79	0			
	Utuan	55	31	56.36	32	58.18 (44.1-71.4)	7	12.73 (5.3-24.5)
	Virian	56	42	75.00	3	5.36 (1.1-14.9)		
Gazelle	Kamanaka	100	57	57.00	0			
	Karo	54	31	57.41	1	1.85 (0.1-9.9)		
	Lan	107	58	54.21	20	18.69 (11.8-27.4)	1	0.93 (0.0-5.1)
	Matanaku	102	56	54.90	3	2.94 (0.6-8.4)		
	Mobilim	100	35	35.00	4	4 (1.1-9.9)		
	Napapar1	98	55	56.12	2	2.04 (0.3-7.2)		
	Puktas	100	55	55.00	4	4 (1.1-9.9)		
	Ragaga	100	61	61.00	6	6.06 (2.3-12.7)		
	Ulak	99	52	52.53	3	3.03 (0.6-8.6)		
	Vunapala	102	63	61.76	1	0.98 (0.0-5.3)		
	Warakind	100	52	52.00	7	7 (2.9-13.9)		
	Wuatam	99	25	25.25	2	2.04 (0.3-7.2)		
Pomio	Awatka	100	55	55.00	4	4 (1.1-9.9)		
	Bogotata	82	54	65.85	12	14.81 (7.9-24.5)		
	Buka	81	47	58.02	23	28.4 (18.9-39.5)		
	Bulus	98	55	56.12	19	19.39 (12.1-28.6)		
	Gar	59	40	67.80	0			

	Gumgum	100	45	45.00	6	6 (2.2-12.6)	1	1.0 (0.0-5.5)
	Hoiya	58	36	62.07	15	25.86 (15.3-39.0)		
	Illi	100	48	48.00	0			
	Ivai	98	74	75.51	6	6.12 (2.3-12.9)	1	1.02 (0.0-5.6)
	Karlai	100	46	46.00	17	17 (10.2-25.8)		
	Kaukum	51	25	49.02	13	25.49 (14.3-39.6)		
	Kavudemk	100	56	56.00	12	12.12 (6.4-20.2)		
	Kolai	50	34	68.00	4	8 (2.2-19.2)		
	Lamarian	99	53	53.54	11	11.11 (5.7-19.0)		
	Lat	107	58	54.21	20	18.69 (11.8-27.4)	1	0.93 (0.0-5.1)
	Long	99	36	36.36	14	14.29 (8.0-22.8)	1	1.02 (0.0-5.6)
	Masarau	77	50	64.94	5	6.49 (2.1-14.5)		
	Milim	88	53	60.23	6	6.82 (2.5-14.3)		
	Muu	99	48	48.48	8	8.08 (3.6-15.3)		
	PomComSc	82	47	57.32	0			
	Pulpul	86	54	62.79	6	7.06 (2.6-14.7)		
	Rainut	50	24	48.00	2	4 (0.5-13.7)		
	Riete	100	51	51.00	2	2 (0.2-7.0)		
	Sivauna	49	28	57.14	6	12.24 (4.6-24.8)		
	Tokai	70	45	64.29	16	22.86 (13.7-34.4)		
Rabaul	Tavui	99	57	57.58	0	-		

Supplemental Figures

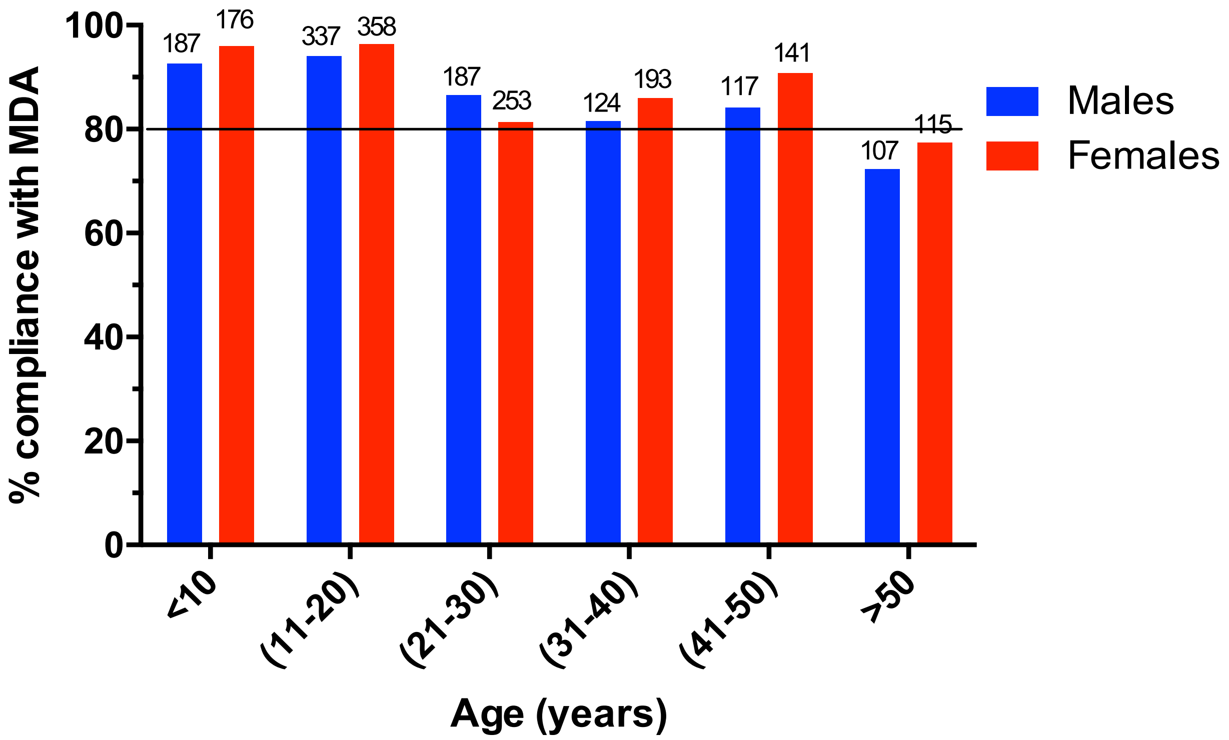

Supplemental Figure 1. Results of a coverage survey of 45 clusters and 450 households representing 2,598 children and adults. The numbers above the bars represent the sample size for each stratum and sex. The dashed line indicates the 80% recommended coverage goal for a highly efficient MDA.

Supplemental Methods

Geostatistical model for selecting clusters at 1-year post-MDA

We use geostatistical methods to model the number of positively tested cases for LF, Y_i, out of n_i sampled individuals at the i-th village location x_i. We then assume that conditionally on spatial Gaussian process $S(x_i)$, the Y_i follow a set of mutually independent Binomial variables with logit-link function. Let $p(x_i)$ denote the probability of a positive test for an individual living in a village at location x_i. The linear predictor is then expressed as

$$\log\left\{\frac{p(x_i)}{1-p(x_i)}\right\} = \beta_0 + \beta_1 d_1(x_i) + \beta_2 d_2(x_i) + S(x_i)$$

where: $d_1(x_i)$ is a covariate which quantifies the environmental exposure to LF at location x_i by combining information on relative humidity, annual precipitation, annual temperature, elevation and distance from the sea using principal component analysis; $d_2(x_i)$ is the socio-economic impact (SEI) score. The rationale for the inclusion of $S(x_i)$ is to account for unmeasured risk factors that, in addition to $d_1(x_i)$ and $d_2(x_i)$, affect LF prevalence. We assume $S(x_i)$ to be an isotropic and stationary Gaussian process with exponential covariance function given by

$$Cov\{S(x_i), S(x_j)\} = \sigma^2 exp\{-||x_i - x_j||/\varphi\}$$

where σ^2 is the variance of the spatial process $S(x_i)$, $||x_i - x_j||$ is the distance, in km, between location x_i and x_j and φ is a scale parameter which regulates how fast the spatial correlation decays to zero for increasing distance $||x_i - x_j||$.

We fitted the model given in the above equation using Monte Carlo maximum likelihood methods included in the PrevMap package [17]. After fitting the geostatistical model described above, we obtained the predictive probability of exceeding 2% prevalence thresholds (or $EP_{2\%}$) at each of the 49 sampled clusters and 699 unsampled clusters. Interpreting the exceedance probabilities $EP_{2\%}$ is: values of $EP_{2\%}$ close to 0 indicate a high probability that prevalence is below 2%; values of $EP_{2\%}$ close to 1 indicate a high probability that prevalence is above 2%; and, if values of $EP_{2\%}$ are close to 50%, this corresponds to the highest level of uncertainty, with an equal probability of exceeding or not the 2% threshold. To identify locations that were more likely to have a higher than 2% prevalence baseline, we proceed through the following iterative steps: i) Set the minimum value of $EP_{2\%}$ say α, used to include new clusters in the sample to 95%, ii) among the 49 sampled clusters, select those for which $EP_{2\%} > \alpha$. If a Ward is represented by more than one village, take only the village with the highest $EP_{2\%}$. Denote this final set of clusters as S_1, iii) among the 699 sampled clusters, select those for which $EP_{2\%} > \alpha$. If a Ward is represented by more than one village, take only the village with the highest $EP_{2\%}$. Denote this final set of clusters as S_2, iv) if the sum of the size of S_1 and S_2 exceeds 50, then increase α to $\alpha^{new} = \alpha + 1/100$; if the sum of the size of S_1 and S_2 is below 50, then decrease α to $\alpha^{new} = \alpha - 1/100$; repeat steps 1 to 3, v) if the sum of the size of S_1 and S_2 is 50, then stop with S_1 and S_2 corresponding to the set of clusters to be sampled. A sample size of 50 clusters was selected as the upper limit in the model comparable to the number of clusters surveyed at baseline.
